# A matter of time or timing: longitudinal analysis of sensitive periods for linear running speed performance and development in elite youth soccer

**DOI:** 10.5114/biolsport.2026.161105

**Published:** 2026-04-20

**Authors:** Maros Kalata, Jakub Lukavsky, Diogo V. Martinho, Craig A. Williams, Jan M. Konarski, Frantisek Zahalka, Robert M. Malina, Tomas Maly

**Affiliations:** 1 Faculty of Physical Education and Sport, Charles University, Prague, Czech Republic; 2 SK Slavia Prague, Prague, Czech Republic; 3 CIPER, Faculdade de Ciências do Desporto e Educação Física, Universidade de Coimbra, Coimbra, Portugal; 4 CIPER, Faculdade de Motricidade Humana, Universidade de Lisboa, Cruz-Quebrada-Dafundo, Portuga; 5 Children’s Health and Exercise Research Centre, Faculty of Health and Life Sciences, University of Exeter, Exeter, United Kingdom; 6 Theory of Sports Department, Poznań University of Physical Education, Poznań, Poland; 7 Department of Kinesiology and Health Education, University of Texas at Austin, Austin, TX, United States; 8 School of Public Health, University of Louisville, Louisville, KY, United States; 9 Department of Performance, AC Sparta Prague, Prague, Czech Republic

**Keywords:** Biological maturation, Sprinting performance, Talent development, Adolescent athletes, Growth status

## Abstract

The objective of the study was to characterize variability in the development of maximal running speed among elite youth soccer players by evaluating its non-linear trajectory relative to both chronological age and biological maturation, and to determine how these trajectories vary relative to estimated maturity timing. A mixed-longitudinal sample of 117 male youth soccer players (10.7–15.9 years, 461 observations) was considered. Maximal running speed was measured via a 20-meter flying sprint. Biological maturity status was estimated as the percentage of predicted adult height (%PAH), and players were classified as early, average, or late maturing. Non-linear developmental trajectories were analyzed using Generalized Additive Mixed Models. Relative to estimated biological maturity status, a consistent peak rate of speed improvement at ~94.4% PAH was noted and it was preceded by a phase of accelerated improvement between ~87.6–90.7% PAH. However, relative to chronological age, the timing of the accelerated developmental phase was related to estimated maturity timing; early and average maturing players experienced this interval of accelerated gains at a significantly younger age than late-maturing peers, who showed no sustained period of acceleration. Maximal running speed performance in youth soccer players is related to biological maturity status rather than chronological age. The identified phases of accelerated improvement represent developmentally meaningful periods that may help contextualize individual differences in the development of sprinting performance and support maturity-based approaches to long-term athlete development, talent identification, and performance monitoring.

## INTRODUCTION

Maximal running speed is a key indicator of performance among youth soccer players [1, 2]. Despite a similar neurophysiological basis, previous research has noted differences and independence among components of speed such as acceleration, maximal running speed, agility and kick speed in young elite soccer players [3]. Although speed improves during childhood, its development is nonlinearly influenced by the process of biological maturation and neuromuscular changes during adolescence such as the stabilization of motor control and strength asymmetries which can fluctuate during the growth [4, 5, 6, 7]. By inference, the development of sprinting performance may be better understood by examining periods during which performance changes occur at an accelerated rate as part of growth and biological maturation [8, 9].

The development of sprinting speed across childhood and adolescence involves two broad phases related to neuromuscular maturation and somatic growth. The first, typically occurs prior to puberty and is characterized by maturation of the central nervous system associated with the acquisition and refinement of movement patterns and coordination that control sprinting speed [10, 11]. The second phase occurs during adolescence, specifically around the interval of peak height velocity (PHV), when rapid somatic growth and biological maturation lead to increases in limb length, muscle mass and strength, thereby enhancing the ability to apply previously acquired coordination to produce maximum force and speed [7]. Significant linear speed improvements during this interval are related to accelerated linear growth and increased muscle mass and lower limb strength [6, 12], which are associated with two key determinants of maximal running velocity (hereafter referred to as maximal running speed) – greater ground reaction forces and longer stride lengths [13].

The development of maximal running speed during puberty and after PHV is also inextricably linked to the enhancement of the stretchshortening cycle (SSC) function [14]. With age and biological maturation, the ability to efficiently utilize elastic energy stored in muscles and tendons improves and directly contributes to faster and more powerful force production, both of which are crucial for sprinting [14, 15]. These maturational changes provide a physiological context within which strength- and power-oriented training modalities, such as plyometric and resistance-based exercises, are commonly implemented in youth sport [16, 17].

Although the existence of discrete “windows of opportunity“ remains debated, empirical support, particularly for speed, often faces criticism due to a lack of robust studies and significant inter-individual variability in the timing and tempo of biological maturation [8, 10, 18, 19]. Allowing for genetic and environmental factors [20] and the risk of overemphasizing specific developmental periods at the expense of continuous development [21], it is generally proposed that periods of accelerated sprinting performance change should be viewed as a guide within a complex, non-linear developmental process [22].

In the context of training, evaluating and identifying talent among young soccer players, it is, therefore, essential to consider not only chronological age (CA) but also to critically evaluate biological maturity status, which is often viewed in the context of variation in maturity timing, i.e., early, average and late [5, 8]. On average, early maturing youth attain developmental milestones such as PHV at an earlier chronological age than late maturing peers [12]. Overlooking this variation may lead to the misjudgment of potential, as late maturing youth ordinarily exhibit lower current levels of sprinting performance associated with the later stages of biological development and not due to less talent [6, 23]. Thus, monitoring maturity status may allow for more equitable talent identification, prevent premature de-selection of late maturing youth, and support developmentally informed approaches to long-term athlete development beyond reliance on chronological age alone [8, 21]. Importantly, such an approach also supports talent development by helping to contextualize current sprinting performance levels and anticipated future rates of improvement relative to maturity timing, rather than short-term performance alone.

In the context of the preceding overview, this study focuses on characterizing the developmental trajectories of sprinting performance (maximal running speed) among U12 through U16 soccer players. The objective is to explore how phases of accelerated sprint performance development are expressed relative to chronological age and estimated biological maturity status.

## MATERIALS AND METHODS

### Participants

The sample included 117 youth soccer players from the academy of Slavia Prague academy in Prague. Data were collected longitudinally over five competitive seasons spanning 2020 to 2025; the mixed-longitudinal data included a total of 461 observations. Seven players (6.0%) were measured once, while the number of repeated measurements of other players varied: 17 (14.5%) twice, 17 (14.5%) three times, 37 (31.6%) four times, 20 (17.1%) five times, 13 (11.1%) six times, 5 (4.3%) seven times, and 1 (0.9%) eight times. The mean number of observations was approximately 4 per player. CA during the interval of the observations ranged from 10.7 to 15.9 years. All players were involved in structured training and a competition schedule characteristic of elite youth academies, including 3–5 training sessions per week (90–120 minutes per session) and one competitive match per week. Training history and formal participation in soccer were recorded for each player. The study was approved by the Institutional Ethics Committee of the Charles University, Faculty of Physical Education and Sport, Approval No. 238/2019, and complied with the Declaration of Helsinki. All players were fully informed about the aims of the study and the testing procedures. Written informed consent was obtained from the parents or legal guardians, and assent was obtained from the players themselves.

### Maturity Status

Biological maturity status was estimated as the percentage of predicted adult height (%PAH) attained at the time of observation, i.e., each measurement occasion [24]. Based on z-scores for %PAH at initial observation, players were classified as early, average or late maturing relative to age- and sex-specific means and standard deviations for the UK 1990 growth reference [25]. Players with a Z-score within ± 0.5 standard deviation (SD) of the group mean were classified as average, while a Z-score less than -0.5 SD and greater than +0.5 SD classified players as late and early maturing, respectively. The ± 0.5 SD criterion was consistent with previous research defining maturity status among youth athletes [26, 27, 28]. Players were classified by maturity status as follows:

– Late: n = 18 players (71 observations; mean 4 obs/player)
– Average: n = 52 players (221 observations; mean 4 obs/player) –
– Early: n = 47 players (169 observations; mean 4 obs/player).

### Maximal Running Speed

Sprinting performance was assessed using a 20-meter flying sprint (ms20F), which is accepted as a field-based indicator of maximal running speed. The flying sprint has good within-session reliability in elite youth soccer players, with reported intraclass correlation coefficients of about 0.90 [29]. In highly trained youth soccer players, a 40-m sprint has been shown to be appropriate among youth in U12–U18 competitive age groups, as maximal sprinting speed is typically attained within this distance and with a high proportion of players reaching maximal speed between 20 m and 30 m [30]. Its validity as a measure of maximal running speed has also been confirmed by strong correlations with in-game performances and GPS data, highlighting its relevance for important neuromuscular capabilities [31]. Prior to testing, all players performed a standardized warm-up protocol consisting of general aerobic activity, dynamic stretching and sprint drills. Maximal running speed was measured as the time required to complete a 20 m distance following a 20 m flying start. Photocells (Microgate, Bolzano, Italy) placed at a height of 0.75 m at the 0 m and 20 m marks of the timing zone, recorded sprint times with an accuracy of 0.01 s. Players were instructed to sprint as fast as possible from the start of the run-up and to continue this maximal effort through the 20 m. Each player performed two trials, with a 5-minute rest between sprints; the faster time was retained for analysis.

### Statistical analysis

Developmental changes in sprint performance (ms20F) were evaluated with Generalized Additive Mixed Models (GAMMs) described by Wood [32]. This approach is an extension of generalized linear models and is specifically suited for analyzing hierarchically structured data, i.e., repeated measures within individuals, and allows for flexible modeling of non-linear developmental trajectories [33].

To analyze developmental trajectories of sprint performance, three generalized additive mixed models (GAMMs) were used to address potential multicollinearity between CA and estimated biological maturity status (%PAH) by modeling each separately or interactively:

Model 1 assessed ms20F performance relative to %PAH, the indicator of biological maturity status, as follows:

ms20F~s(%PAH,k=10)+s(ID,bs=“re”).

Model 2 assessed ms20F development relative to CA as follows:

ms20F~s(CA,k=6)+s(ID,bs=“re”).

Model 3 addressed the influence of CA relative to maturity status (late, average or early) as follows:

$$\begin{array}{c} \text{ms}20\text{F}\sim \text{maturity}\_\text{timing}\_\text{group}\\ +\text{s}(\text{CA},\text{by}=\text{maturity}\_\text{timing}\_\text{group},\text{ k}=5)\\ +\text{s}(\text{ID},\text{bs}=“\text{re}”) \end{array}$$

allowing for group-specific intercepts and developmental curves.

An interaction model considering %PAH with maturity timing groups (Model 4) was also explored, but was not retained as it indicated no statistically significant group-specific effects. In each model, s(ID, bs = “re”) random intercepts for individual players accounted for repeated measures. A Gaussian error distribution with an identity link function was assumed, and parameters were estimated using Restricted Maximum Likelihood (REML). The basic dimension *k* for smooth terms was chosen a priori (10 for s(%PAH) in Model 1, 6 for s(CA) in Model 2, and 5 for group-specific s(CA) smooths in Model 3) to accommodate sufficient flexibility and to prevent overfitting.

Model fit was estimated using the adjusted coefficients of determination (R^2^) and the REML score. The significance of non-linear smooth terms was evaluated based on F-statistics and associated pvalues. To characterize the dynamics of developmental changes in more detail, first (rate of change) and second (change in the rate of change) derivatives of the population-average trajectories were numerically derived from both models with respect to the relevant predictor (%PAH or CA). Intervals of performance improvement (i.e., where the 95% CI of the first derivative was entirely negative), acceleration of the improvement rate (i.e., where the 95% CI of the second derivative was entirely negative), or deceleration of the improvement rate (i.e., where the 95% CI of the second derivative was entirely positive) were identified using the 95% confidence intervals of the respective derivatives.

**FIG. 1:**
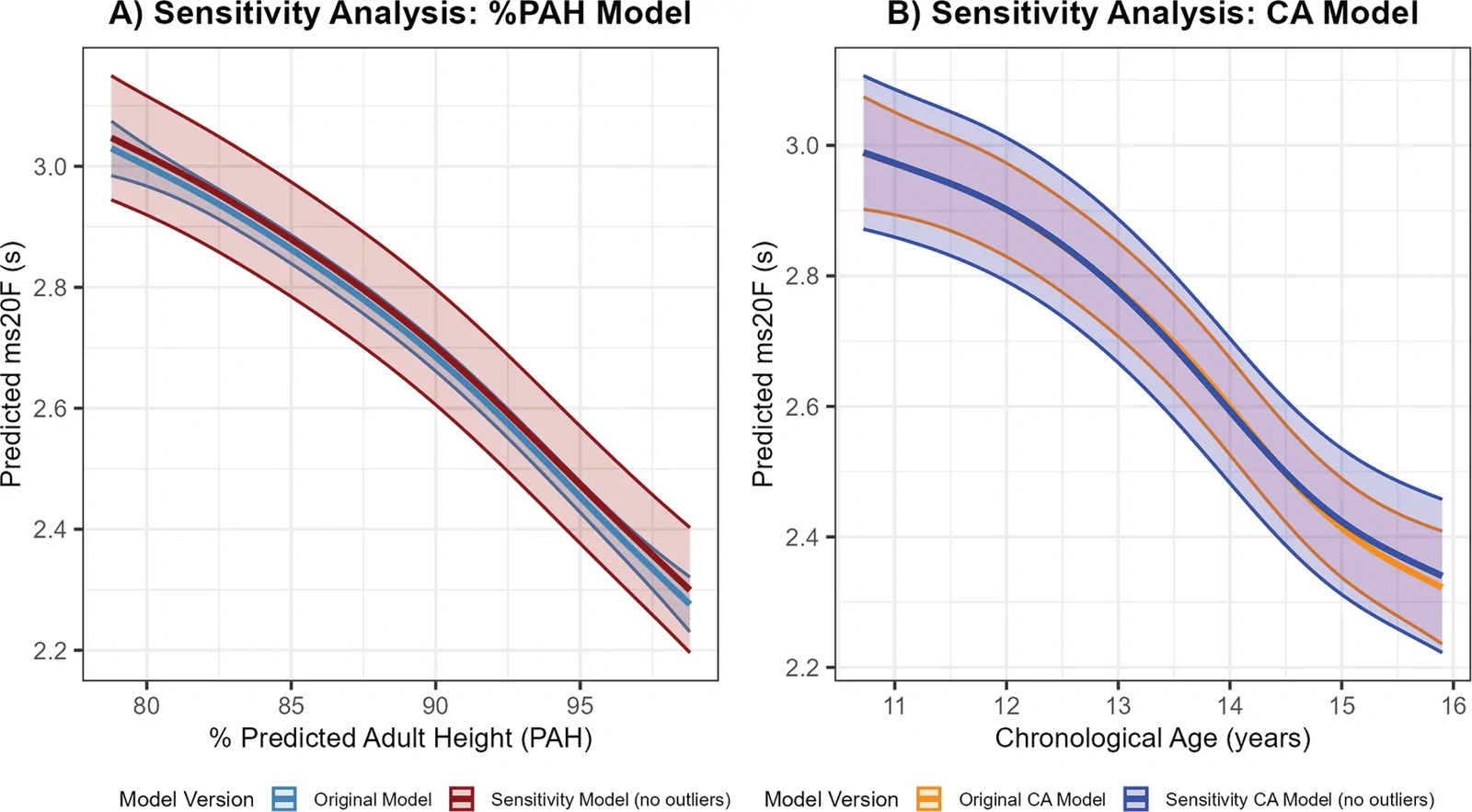
Sensitivity analysis of developmental trajectory models for maximal running speed based on the percentage of predicted adult height (A) and chronological age (B).

Model diagnostics included verification of several assumptions: adequacy of the basic dimension (*k*) for smooth terms (gam.check function), visual assessment of residual normality (Q-Q plots, histograms), and homoscedasticity (plots of residuals versus fitted values). Potential outliers were identified based on the diagnostics. To assess their influence, a sensitivity analysis was conducted by re-fitting the models after temporarily removing the identified observations. The results (Fig. 1A, Fig. 1B) confirmed that the shape of the predicted developmental curves and key aspects of the developmental dynamics were robust and not substantially influenced by these observations. Thus, all data were retained in the final models presented. All analyses were performed using R (version 4.5.0; R Core Team, 2025) and the mgcv package (version 1.9-1; Wood, 2017) [32]. The level of statistical significance was set a priori at α = 0.05 (corresponding to a 95% confidence level).

## RESULTS

**FIG. 2:**
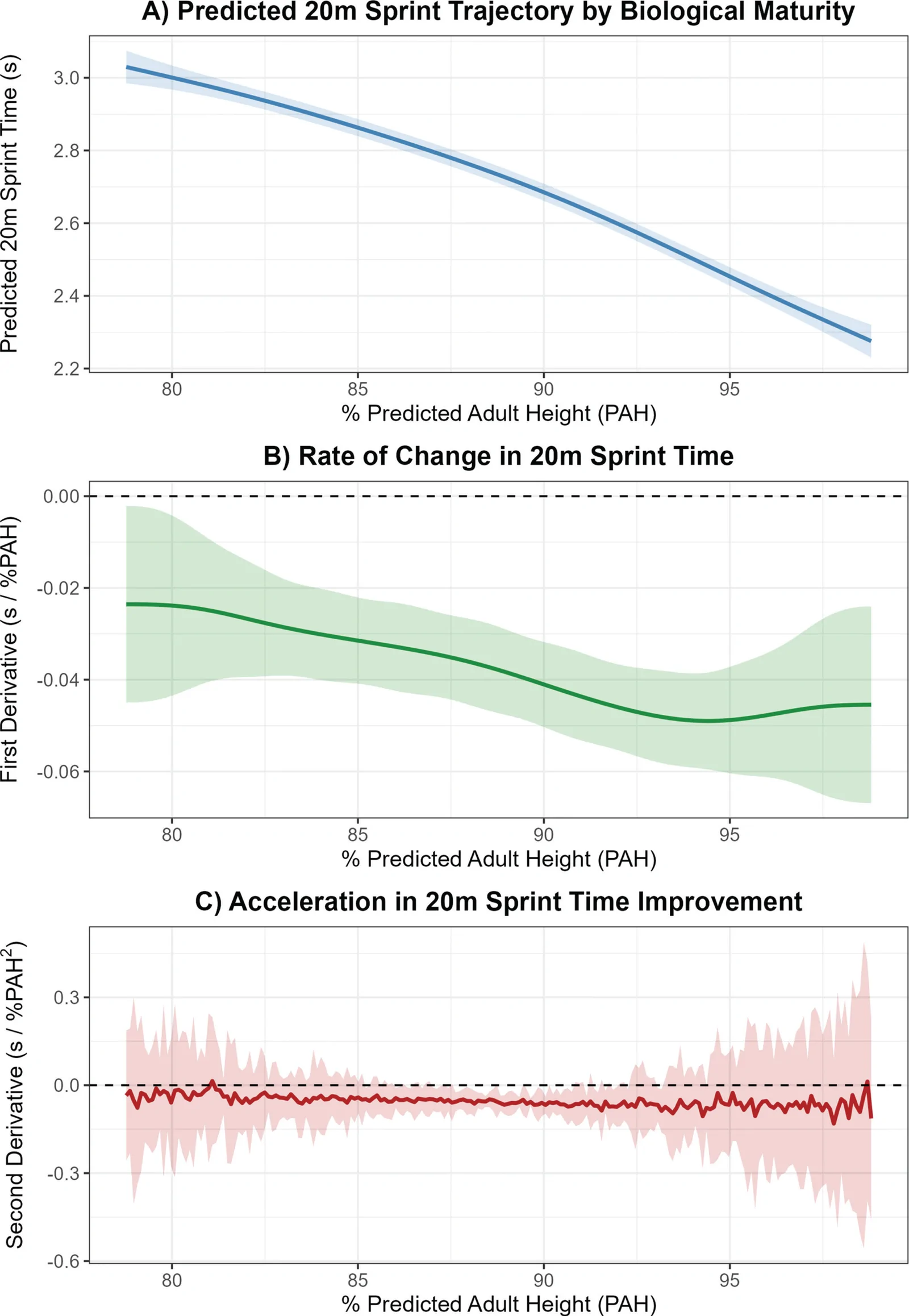
Developmental trajectory and rate of change in maximal running speed (ms20F) relative to the percentage of predicted adult height (%PAH).

Model 1 (adj. R^2^ = 0.89; REML = -409.21) indicated a statistically significant non-linear relationship between %PAH and ms20F (for s(PAH): edf = 3.47; F = 304.21, p < 0.001). The developmental trajectory for observed %PAH (range of 78.77–98.80%) predicted an average ms20F improvement of approximately 24.9% (a decrease of 0.75 s, from ~3.03 s to ~2.28 s; Fig. 2A). Dynamic analysis showed a continuous and statistically significant improvement in ms20F across the entire range of %PAH. The peak rate of improvement occurred at approximately 94.4% PAH, with a rate of -0.05 s (95% CI: -0.06 to -0.04 s) per 1% increase in %PAH (Fig. 2B). Periods of statistically significant acceleration in the rate of improvement were noted between ~85.2–93.2% PAH, with the most pronounced phases between ~87.6–88.6% PAH and ~89.5–90.7% PAH (Fig. 2C). Following the inflection point at ~94.37% PAH, the rate of improvement decreased, although no statistically significant period was identified.

**FIG. 3:**
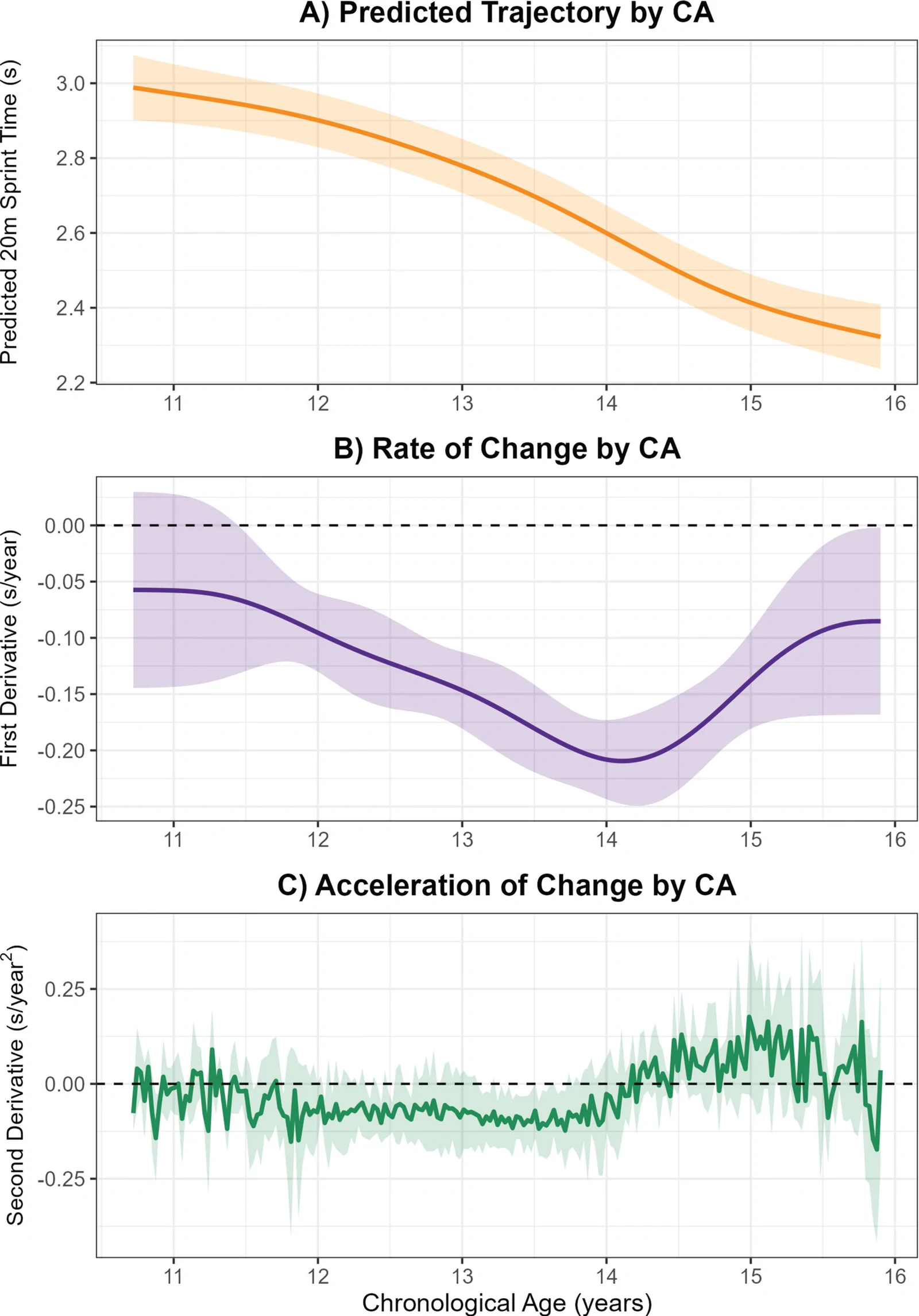
Developmental trajectory and rate of change in maximal running speed (ms20F) relative to chronological age (CA).

Model 2 (adj. R^2^ = 0.89; REML = -391.3) indicated a statistically significant non-linear relationship between CA and ms20F (for s(CA): edf = 4.25; F = 257.16, p < 0.001). The developmental trajectory with CA (10.72–15.90 years) predicted an average ms20F improvement of approximately 22.3% (a decrease of 0.67 s from ~2.99 s to ~2.32 s; Fig. 3A). Dynamic analysis indicated statistically significant improvement in ms20F from 11.45 years of age to the end of the observed interval (15.90 years). The peak rate of improvement occurred at approximately 14.10 years, with a rate of -0.21 s/year (95% CI: -0.25 to -0.17 s/year) (Fig. 3B). Intervals of statistically significant acceleration in the rate of improvement were noted between ~13.11–13.71 years (Fig. 3C), and following the inflection at ~14.10 years, the rate of improvement declined, although the deceleration was statistically significant only at 14.52, 14.55, 14.75, 15.17, and 15.20 years.

Model 3 considered the development of ms20F relative to CA and maturity timing classified as late, average and early; the model indicated distinct developmental patterns for youth in each maturity group (adjusted R^2^ = 0.88; REML = -386.55).

**FIG. 4:**
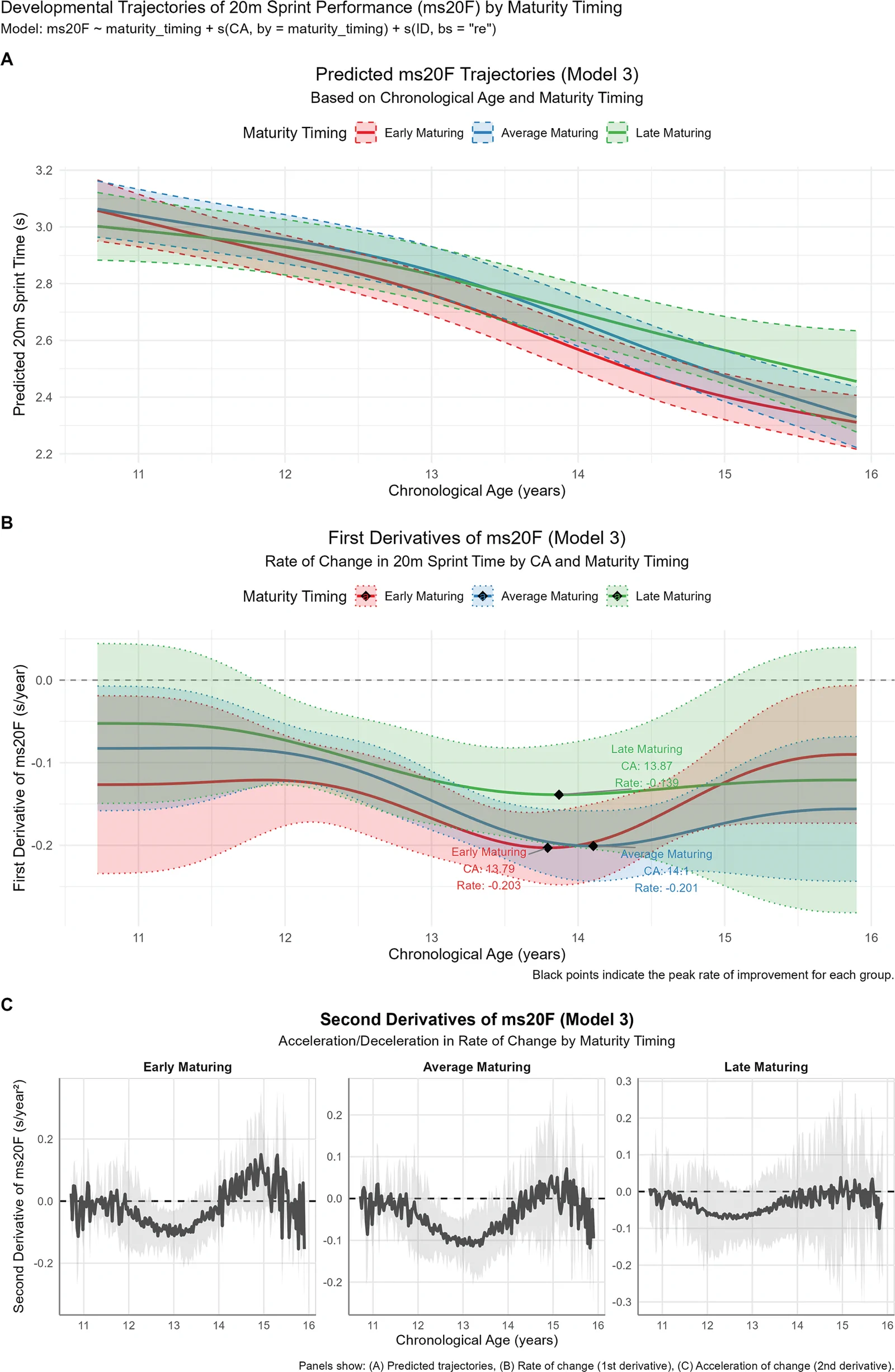
Developmental trajectories and rates of change in maximal running speed (ms20F) by maturity timing group.

The predicted ms20F trajectories (Figure 4A) showed that late maturing players initially exhibited the fastest 20 m sprint times at about 10.7 years, but subsequent performances improved the least over time, resulting in the slowest times by 15.9 years. Conversely, early maturing players, despite a slightly slower start than late maturing peers at 10.7 years, demonstrated substantial and consistent improvement in sprint times and attained the fastest sprint times by the end of the observation interval. Average maturing players displayed an intermediate trajectory.

In contrast, the rate of ms20F improvement (1^st^ derivative; Figure 4B) varied among maturity groups. Early maturing players showed significant improvement across the entire age range 10.7–15.9 years, with a peak rate of improvement (-0.20 s/year) at approximately 13.79 years. Average maturing players also improved significantly across the interval, reaching a peak rate (-0.20 s/year) slightly later, 14.10 years. Of interest, the estimated rate of improvement in average maturing players did not differ statistically from early maturing players across the CA range. Late maturing players, in contrast, showed a shorter period of significant improvement (approximately 11.81–15.01 years) and a lower peak rate of improvement (-0.14 s/year) at 13.87 years. Late maturing players also improved at a significantly slower rate than early maturing players between approximately 13.11 and 14.08 years, and at a significantly slower rate than average maturing players between approximately 13.48 and 14.34 years.

The analysis of acceleration in ms20F improvement (2^nd^ derivative; Figure 4C) showed that early maturing players experienced a significant acceleration in the rate of improvement between approximately 12.57 and 13.24 years, while average maturing players showed a similar interval of significant acceleration between about 12.52 and 13.48 years. In contrast, no sustained period of statistically significant acceleration in the rate of improvement was noted among late maturing players.

## DISCUSSION

The present study provides a novel evaluation of maximal running speed development (MSD) among youth male soccer players, with a specific focus on identifying phases of accelerated change during adolescence when key changes in performance occur and quantifying the rate of these changes. Periods of accelerated sprinting performance were evaluated in the context of both estimated biological maturity status (%PAH) and CA. A key observation was that, although biological maturity status may indicate comparable developmental milestones for performance acceleration in the maturity timing groups, the CAs at which these phases of accelerated development occurred varied with estimated maturity timing, i.e., among youth classified as early, average, or late maturing. Specifically, peak rates of improvement and phases of greatest improvement in MSD were attained at a younger CA among early and average maturing players compared to their late-maturing peers (Figure 4A). This observation underscores the significance of variation in estimated maturity timing; specifically as youth in the different maturity timing groups traversed key phases of speed development at different CAs, even at comparable levels of estimated %PAH.

The findings of the present study are central to discussions of and contrasts between the Long-Term Athlete Development (LTAD) [34] and the Youth Physical Development (YPD) [11] models. The identification of biologically timed phases of accelerated sprinting performance change is consistent with the concept of developmental “windows of opportunity” in the LTAD model [34]. At the same time, the present results strongly support emphasis on development as a continuous, non-linear and highly individualized process implicit in the YPD model [11].

A unique contribution of this study is the clarification of how these two talent development models coexist. The present findings support the existence of developmentally timed windows of opportunity, but the timing is anchored to estimated biological maturity and not chronological age. This observation explains why approaches based solely on CA are limited, as players pass through this critical window at different CA depending on variation in maturity timing of individuals. This observation is also consistent with an emerging and integrated view supported by other evidence [7] and moves beyond the “either/or” dichotomy. This perspective suggests that while foundational qualities should be developed continuously, as advocated by the YPD model, practitioners should consider biologically maturity-timed intervals of accelerated MSD as periods of developmental relevance.

The evaluation of MSD in relation to predicted biological maturity status in the present study, noted statistically significant improvement across the CA interval of estimated biological maturity status considered (78.8% to 98.8% PAH), which was consistent with the observations of Radnor et al. [7]. The peak rate of improvement in sprinting performance was observed at approximately 94.4% PAH in the present study, which was consistent with previous research highlighting the interval of PHV or shortly thereafter as critical for substantial gains in sprinting speed [6, 7]. The significant increase in speed at the time of and after PHV was attributed to increased stride length and the stabilization of stride frequency and contact time [6]. The marked improvement at about 94.4% PAH in the present study thus likely reflected the phase in somatic growth where growth in leg length is largely complete, and athletes can more effectively translate the increased adolescent growth in muscle mass and strength, both characteristic of later maturational stages, into enhanced maximal running speed mechanics [19]. Importantly, these changes reflect maturity-driven performance development rather than a direct measure of training responsiveness. These findings are also consistent with interventional studies, such as Asadi et al. [35], which noted that while plyometric training was effective across all estimated maturity stages, the most significant performance adaptations were observed in post-PHV athletes.

Results of the present analysis also indicated statistically significant phases of accelerated MSD development at an estimated %PAH of 85.2% and 93.2%, with the most intense increases occurring between ~87.6–88.6% and ~89.5–90.7% of estimated %PAH. These phases of accelerated development, which precede the peak rate of improvement, highlight periods of pronounced developmental change within the maturation process. The observation of an accelerated development phase, rather than a performance decline, aligns strongly with recent longitudinal observations. Radnor et al. [7], for example, noted that the largest improvements in sprinting performance over an 18-month interval occurred among athletes who progressed from predicted pre- to post-PHV maturity status. This recent perspective is apparent in the study of Bath et al. [36], which noted that PHV typically occurred at approximately 91–92% PAH among elite youth soccer players, consistent with the acceleration phases noted during the early pubertal stages immediately preceding PHV in the present study.

These early adaptations are likely associated with a rapid improvement in the ability to adjust to changing body biomechanics. Specifically, and as demonstrated by Rumpf et al. [37], advanced biological maturity status is associated with increased vertical and leg stiffness, which is crucial for optimizing SSC function. Concurrently, this interval is associated with the development of key neuromuscular characteristics, specifically improved intermuscular coordination and motor control, which enables more effective force transfer and adaptation to changes associated with physical growth [8]. Overall, the evidence for continuous neuromuscular enhancement helps to explain the findings of the present study indicating a continuous improvement in sprint performance throughout the process of adolescent maturation, which contrasts with earlier research [12, 38]. The earlier studies, identified a temporary decline in performance, an observation that was often attributed to ‘adolescent awkwardness’ during the growth spurt. This discrepancy may reflect differences in cohort characteristics, methodological approaches, and/or broader changes in youth sport environments that influence how adolescents adapt to rapid growth and related changes in motor control. An alternative explanation proposes an increasing association of the phenomenon of ‘adolescent awkwardness’ not with linear sprinting, but rather with more complex, multi-directional tasks, associated with performance discrepancies in activities such as soccer dribbling and sideways jumping [39, 40].

Following the interval of most rapid improvement (at ~94.4% PAH), the rate of improvement in sprinting performance began to decline, although the deceleration was not statistically significant. Considering the reported variability in the timing of PHV when expressed as %PAH, the peak observed at ~94.4% PAH can be interpreted as occurring in close temporal proximity to this maturational landmark. This observation is consistent with findings of Philippaerts et al. [12] among youth Flemish soccer players, i.e., following peak development of running speed (30 m sprint with a flying start) during the interval of PHV, a performance plateau persisted for 12 to 18 months after PHV. A possible explanation for such a plateau or perhaps deceleration in performance is the attainment of the limits of physiological and/or biomechanical adaptations driven primarily by the processes of growth and maturation. It is also possible that further improvements beyond this stage require factors beyond maturation per se. Alternatively, the trend may simply reflect the natural course of the developmental curve, where a period of rapid growth is followed by a phase of stabilization or more modest improvement [5].

The present study highlights a potential dual role of maturity timing. Although maturity timing often becomes redundant in models based on biological maturity status as players are commonly grouped relative to an indicator of biological timing, variation associated with maturity timing emerges as a decisive factor when development is plotted relative to CA. The analysis of developmental trajectories in the present study confirmed the well-documented pattern [41], i.e., initially faster developing late maturing youth were progressively surpassed by their early-maturing peers. A more specific insight is provided by the rate of improvement; while early and average maturing youth showed a statistically similar peak rate, the peak occurred sooner in early maturers. Of relevance (and also often overlooked), at the end of the observation period (about 15.9 years), the rate of improvement for early maturers was declining, while average maturers maintained a higher tempo of improvement. These contrasting trajectories are consistent with established growth and maturation models, whereby early maturing boys experience earlier and more pronounced puberty-related changes in muscle structure, neuromuscular coordination, and force production, which may underline the steeper improvements in sprint performance observed in this group compared with later maturing peers [14, 19]. This observation was also corroborated by in-match observations; Parr et al. [42] noted, for example, that while advanced maturation among U14 players was specifically associated with greater high-speed running (HSR), variation in maturity status no longer contributed to the variance in any of the running performance indices among U15/16 players. In contrast, late maturing players not only had a lower overall rate of improvement, but the improvement was also significantly slower during the critical period between 13 and 14 years of age.

Of interest, a more precise definition of accelerated developmental change was apparent in the analysis of the acceleration of improvement. Among early maturing players, this window was shorter compared to average maturing peers. By inference, because early maturing youth enter this phase with an already superior performance level, their capacity for accelerated gains may be more rapidly reached. Among late maturing players, in contrast, no sustained period of statistically significant acceleration was identified. This finding was generally consistent with principles that emphasize that biological windows are linked to maturation and not the calendar [11]. From the perspective of talent development, these findings emphasize that differences in sprint performance at a given chronological age may reflect maturity-related timing effects rather than long-term performance potential.

### Limitations

The results of the present study should be considered in the context of several limitations, which may provide avenues for future research. First, it must be acknowledged that the indicator of maturity status was based on %PAH attained at the time of observation, in contrast to skeletal maturity or pubertal status [19, 43]. Nevertheless, %PAH is a validated, reliable and widely-used method in field-based research, particularly among elite youth soccer players [44, 45]. Second, player attrition associated with the academy environment implies that not all youth were tracked across the entire maturity spectrum. Future research with more complete longitudinal data may refine the boundaries of the identified windows. Finally, the analysis focused on sprint time as the primary performance outcome. Although it is a critical measure of performance, sprint time per se does not elucidate the underlying mechanisms associated with the observed changes. Future investigations should attempt to integrate more detailed metrics, e.g., kinematic measures of stride length and stride frequency, and force-time characteristics based on force plates, to provide a more comprehensive understanding of the development of sprint performance during the intervals of accelerated developmental changes associated with adolescence among soccer players.

## CONCLUSIONS

The period of accelerated improvement in maximal running speed was consistent when tracked relative to an estimate of biological maturity status based on %PAH, but its timing relative to CA was influenced by individual differences in maturity timing. Of importance, the results indicated preparatory phases of accelerated improvement (between ~87.6–90.7% PAH) that precede the ultimate peak rate (~94.4% PAH). The findings support an integrated model where continuous development is enhanced by considering biologically timed intervals of accelerated development during adolescence. In this context, a maturity-informed interpretation of sprint development trajectories may be particularly valuable for talent identification and longterm talent development and may help to distinguish transient maturity-related advantages from longer-term developmental potential.
